# Supplementary material for: Water Effective Diffusion Coefficient in Dairy Powder Calculated by Digital Image Processing and through Machine Learning Algorithms of CLSM Micrographs
Source: Foods. 2023 Dec 27;13(1):94. doi: 10.3390/foods13010094 (PMC10778944; doi:10.3390/foods13010094)
Supplement: Supplementary file 1 [file foods-13-00094-s001.zip › ML_Supplementary.pdf]

Supplementary Material for 2D Water Diffusion Model in Dairy Powders with Digital Image Processing of CLSM micrographs

This supplementary document provides additional resources and detailed information supporting the research presented in our article. It includes references to the source code used for image analysis and machine learning models, as well as further elaborations on methodologies and data.

1. Source Code Reference

The computational analyses and machine learning models discussed in our research were developed using custom-written code. The source code is publicly available for review, replication, and further research. It can be accessed through the following GitHub repository:

- **GitHub Repository for Powder Dissolution Analysis**: https://github.com/mrYush/powder_dissolution

This repository contains all the scripts and code modules used for image processing, data analysis, and machine learning model training and validation. The repository includes:

- Scripts for image preprocessing and segmentation.
- Machine learning models for particle identification and analysis.
- Data processing scripts for calculating effective diffusion coefficients.
- Additional utility scripts for data visualization and statistical analysis.
